# Dietary diversity is related to socioeconomic status among adult Saharawi refugees living in Algeria

**DOI:** 10.1186/s12889-017-4527-x

**Published:** 2017-07-03

**Authors:** Marianne Sandsmark Morseth, Navnit Kaur Grewal, Ida Sophie Kaasa, Anne Hatloy, Ingrid Barikmo, Sigrun Henjum

**Affiliations:** 1 Faculty of Health Sciences, Oslo and Akershus University College, Postbox 4, St. Olavs plass, 0130 Oslo, Norway; 2 FAFO, PO Box 2947, Toyen, NO-0608 Oslo, Norway

**Keywords:** Dietary diversity, Saharawi refugee population, Socioeconomic status, WAMI

## Abstract

**Background:**

There is limited knowledge about dietary quality among the adult population in low- and middle income countries (LMICs). This study aims to describe dietary quality among the adult Saharawi refugee population and to investigate whether dietary quality is associated with socioeconomic status.

**Methods:**

In 2014, a cross-sectional survey was carried out in the Saharawi refugee camps, Algeria. A three-staged cluster sampling was performed and 180 women and 175 men, aged 18–82 years, were randomly selected. The dietary intake was assessed by 24-h dietary recall and dietary diversity score (DDS) was calculated. Socioeconomic status was assessed using the WAMI index (sanitation, assets, education and income).

**Results:**

The mean DDS among the total sample was 3.8 ± 1.4 and 2/3 of participant were at risk of low dietary adequacy. The main food groups consumed were starchy staple foods, flesh foods, and dairy. Vitamin A-rich dark green leafy vegetables, nuts and seeds and eggs were the food groups least consumed. The multiple regression model showed a positive association between DDS and the WAMI index (*P* < 0.001) and a negative association between DDS and age (*p* = 0.01).

**Conclusions:**

Low DDS was associated with low socioeconomic status. Programmes to improve the dietary quality among the Saharawi refugees should be implemented.

**Electronic supplementary material:**

The online version of this article (doi:10.1186/s12889-017-4527-x) contains supplementary material, which is available to authorized users.

## Background

Households in low- and middle income countries (LMICs) typically base their diets of few other food groups than their staple foods, resulting in low dietary diversity [1]. Several studies have shown that dietary diversity is a good proxy of dietary quality among people living in both industrialized and developing countries [1–5]. Through the past decades, economic development has resulted in changes in food consumption patterns in LMICs, where western fat and sugar-rich foods tend to replace traditional grains- and fiber-rich foods. These changes in food consumption, named the Nutrition Transition, is found to affect individuals with high socioeconomic status first [6]. The transition is also seen in North Africa and the Middle East where consumption of meat, eggs, dairy, vegetables, nuts, sugar and oil has increased in the past 50 years [7]. Although these changes may be beneficial for dietary diversity, increased intake of western foods is also associated with obesity and non-communicable diseases [8].

Socioeconomic status is a major determinant of healthy diets in high income countries, and studies have indicated that high socioeconomic status may be associated with overall healthier dietary patterns, diet quality and diversity in low- and middle income countries as well [9]. In a review based on data from 33 LMICs, high SES or living in urban areas was associated with beneficial dietary patterns such as higher intakes of protein; unsaturated fat; iron; and vitamins A and C, but also increased intake of saturated fat and cholesterol and low intake of fiber [9]. Dietary diversity may also be seen as a proxy of household resources. A multi-country analysis by Hoddinott & Yohannes (2002) showed that increased dietary diversity was associated with higher income and higher per capita energy availability at household level. The magnitude of the association between dietary diversity and per capita energy availability was higher for non-staple than for staple foods, reflecting socioeconomic constraints to eating a varied diet [10].

Since 1975, refugees from Western Sahara have been living in the Algerian desert. The refugees are dependent on food aid in the resource-poor desert environment. Vulnerable refugees receive monthly basic food rations with dry foods and oil as well as fresh foods such as seasonal fruits and vegetables from international donors [11]. In addition, food is sold in local shops, but the selection, especially of fresh foods, is limited by what is available at the market in the nearest city [11]. Studies among the Saharawi refugee population have shown several challenges related to nutrition and health [12]. A high prevalence of anemia [11] and obesity among women in the camps is symptomatic of double burden of disease with both micronutrient deficiencies and non-communicable diseases present in the same population or even the same households [13]. In the scientific literature, according to our knowledge, there is no information about diet and dietary quality among the adult refugee population. Information about dietary quality may help the Saharawi government to plan for nutrition interventions.

## Methods

### Aim, study design and sample

The present study aimed to assess diet, dietary diversity and the association between dietary diversity and socioeconomic status among the adult population of Saharawi refugees. A cross-sectional survey was carried out in September and October 2014 in five refugee camps near Tindouf, Algeria. At the date of the interview, the median (5, 95 percentile) number of days since receiving the dry food ration was 11 (1, 31), while corresponding numbers for the fresh food ration was 8 (1, 26). The total population in all five camps was estimated to be approximately 165,000. In the present study, the eligible population were all adults, both males and females, above 18 years of age (the oldest aged 82) living in one of the five refugee camps: Smara, El Aiune, Ausserd, Dakla or Boujdor. Inclusion criteria were that the participants were able to answer the questions from the questionnaire and recall their diet from the previous day. Individuals who were sick, bedridden or due to some reason unable to answer questions were excluded from the study.

The sample size was chosen based on an estimated prevalence of an inadequate diet of 50% and an absolute precision of 5% for the 80% confidence interval. Assuming an incomplete sampling from approximately 10% of the participants, we calculated a final desired sample size of 180 men and 180 women, as determined with Open Source Epidemiologic Statistics for Public Health (OpenEpi). Due to the unequal number of inhabitants in the five camps, a probability proportional to size (PPS) method was used to select participants from each camp. We assumed a 50/50 gender balance in all the camps. This resulted in an estimated sample of 51, 51, 38, 23 and 17 persons of each gender from Smara, El Aiune, Ausserd, Dakla and Boujdor, respectively. The final sample size consisted of 355 participants, 175 men and 180 women.

A three-staged cluster sampling was performed. The first stage was to select camps (PPS) and the second stage was to select households. They were randomly selected by tossing a pen in order to decide the direction in which the research team was to drive upon leaving the dispensaries. The team drove toward the boarder of the camp and each seventh household was selected. The third stage was to randomly select one man and one woman from each household. If more than one man or woman in the household was eligible and wished to participate, numbers were assigned to each person. A fieldworker then pointed to a piece of paper with numbers in random order and the person who matched the selected number was included. In households where men were not present, the woman was included and a man in the neighboring household was asked to participate.

### 24-h dietary recall

The dietary intake was assessed by a single 24-h dietary recall. The dietary recalls were conducted by local field workers fluent in English, Spanish and Hassania (the local language). All field workers were trained in interview technique over a 7 days period, including 2 days for conducting a pilot study. The pilot study was performed on a total of eight men and women to practice the 24-h recall interview.

During the 24-h recall, the participating women and men were asked to name all foods and drinks consumed the preceding day as well as the time of consumption. Names of dishes and all ingredients used were written down. The amount of food consumed was registered using household measurements such as a big spoon, a small spoon, a ladle, a cup, a glass and a tea glass. The food and drink consumption was restricted to 15 g for the item to be included in the analysis. If the participant reported to have consumed a food or drink item below 15 g three or more times during the recall day, the total amount of the item was assumed to be above 15 g and included in the analysis. In addition, we registered if the food or drink was 1) received from the food ration, 2) was bought or 3) if the participant had received it as a gift or eaten the food outside the household. Data on meal pattern was also collected.

The dietary diversity score (DDS) was defined as the number of food groups consumed during the recall day. The present paper used the food groups from Minimum Dietary Diversity – Women (MDD –W) introduced in 2014 by the Food and Agriculture Organization of the United Nations [14] and the Food and Nutrition Technical Assistance Project (FANTA) [14]. The MDD-W uses ten food groups (Table 1). According to this indicator, a consumption of at least five food groups out of ten indicates a greater likelihood of meeting micronutrient needs compared to those consuming foods from fewer food groups.

**Table 1:** Food group classification introduced by FAO and FANTA

1. All starchy staple foods	6. Eggs
2. Beans and peas	7. Vitamin A-rich dark green leafy vegetables
3. Nuts and seeds	8. Other vitamin A-rich vegetables and fruits
4. Dairy	9. Other vegetables
5. Flesh floods	10. Other fruits

### Method for measuring socioeconomic status

Information on demographic data and measurements on socioeconomic status were collected through a questionnaire. The WAMI index (**W**ater/sanitation, **A**ssets, **M**aternal education and **I**ncome) described by Psaki et al. [15] was used to assess socioeconomic status. Since the entire population receives purified water from wells, only data on whether or not the household owned an individual latrine was used to calculate points for Water/sanitation. Participants with individual latrines got 4 points whereas those with no or common latrine got 0 points. This resulted in a maximum score of 28, as opposed to 32 in the original WAMI Index [15]. More specifically half a point was awarded per asset (for list of all assets, see Additional file 1: Table S1 – only the assets in bold are included in the index). The 16 assets included were kitchen, cellphone, solar energy, TV, oven, refrigerator, converter, air condition, radio, sitting furniture, car, washing machine, sleeping mattress, laptop, fan and aggregate. Points for education increased gradually from 0 for no education to 8 for higher education. Finally, those with no income got 0 points, those with income up to 30 Euros got 4 points and above 30 Euros 8 points (see specifications Table 5).

### Data processing and statistical analysis

All statistical analyses were performed in SPSS version 22.0. Data are presented as mean and SD for continuous variables or as percentages for categorical variables. All data from the 24-h recalls were registered manually and each food item received a food code. Each food or drink item consumed above 15 g was further ascribed to one of the ten food groups to describe the dietary diversity score. Mean dietary diversity score (MDDS) was used as a dependent variable in multiple linear regression analyses. A theory-based approach was used to select candidate variables for inclusion in the model. The following variables known to influence DDS, together with selected socio-economic variables were included in the initial crude models; age, gender, pregnancy, Body Mass Index (BMI), number of children, marital status, WAMI index (Water/sanitation, Assets, Maternal education and Income), cultivating vegetables and livestock. All covariates showing a linear association (*p* < 0.10) in the crude regression models were included in a preliminary multiple regression model. Excluded variables were reintroduced and those who were still significantly associated in this model (*p* < 0.10) were retained in the final model. Analysis of the residuals was performed in order to examine the fit of the model.

### Ethical clearance

Ethical clearances were obtained from the Saharawi Health Authorities and the Regional Ethics Committee in Norway. Informed written consent was obtained from all participants. The study was conducted according to the guidelines provided in the Declaration of Helsinki.

## Results

Background characteristics are presented in Table 2. Mean age of the participating men and women were 43 (±19) and 40 (±14) years, respectively. Mean BMI of the participating men and women were 22.4 (±4.2) and 27.6 (±5.7) respectively. Five percent of men and 32% of women were obese. Twenty-seven percent of men and 32% of women had no education, while 6% of men and 4% of women had higher education; no significant differences were found between men and women. Only 10% of the participants were cultivating vegetables and 67% had livestock.

**Table 2:** Characteristics of the total sample (*n* = 355), men (*n* = 175) and women (*n* = 180), Saharawi refugee camps, Algeria 2014

Characteristic	Total^a^		Men^a^		Women^a^		*P*-value^*^
	Mean	SD	Mean	SD	Mean	SD	
Age (years)	42	17	43	19	40	14	0.172
Weight (kg)	66.9	14.1	64.0	13.3	67.9	14.7	0.010
Height (cm)	162.9	9.2	169.0	7.7	157.1	6.4	<0.001
Waist (cm)	85.7	13.6	81.5	12.7	89.9	13.3	<0.001
BMI (kg/m^2^)	25.0	5.6	22.4	4.2	27.6	5.7	<0.001
< 18.5	38 (11)		34 (19)		4 (2)		<0.001
18.5–24.9	156 (45)		97 (55)		59 (34)		<0.001
25.0–29.9	89 (25)		35 (20)		54 (31)		0.017
> 30.0	67 (19)		9 (5)		58 (33)		<0.001
Education							
None	106 (30)		48 (27)		58 (32)		0.384
1st-6th grade	82 (23)		33 (19)		49 (27)		0.081
7th–9th grade	95 (27)		50 (29)		45 (25)		0.522
10th–12th grade	56 (16)		34 (19)		22 (12)		0.086
Higher education	16 (4)		10 (6)		6 (4)		0.409
Married	204 (58)		97 (55)		107 (59)		0.511
Livestock^b^	237 (67)		121 (69)		116 (64)		0.347
Cultivating vegetables^c^	35 (10)		22 (13)		13 (7)		0.091

^*^ *P* values for the comparison of men and women

^a^Data are presented as mean and SD for continuous variables or as number of participants and percentages for categorical variables

^b^Goats, sheep, chicken & camels

^c^Tomatoes, herbs, carrots, squash and onions

### DDS and food groups

The mean DDS among men and women was 3.8 (±1.4), with a mean DDS of 3.9 (±1.3) among the women and 3.8 (±1.5) among the men (Table 3). Number of food groups consumed ranged from one to seven, with 32% of the participants having consumed foods from 5 or more food groups. Main food groups consumed were starchy staple foods, flesh foods, and dairy. Vitamin A-rich dark green leafy vegetables, nuts, seeds, and eggs were the food groups least consumed (Table 4). Ninety-seven percent of the women and 95% of men had consumed tea, coffee, and sugary foods and drinks, while the corresponding numbers for oil and fat was 64 and 66%.

**Table 3:** Number of food groups consumed during the 24-h recall (*n* = 354)

Number of food groups	Total, *n* (%)	Men, *n* (%)	Women, *n* (%)
1	9 (3)	6 (3)	6 (2)
2	41 (12)	24 (14)	17 (9)
3	116 (33)	54 (31)	62 (34)
4	73 (21)	32 (18)	41 (23)
5	71 (20)	33 (19)	38 (21)
6	32 (9)	18 (10)	14 (8)
7	12 (3)	7 (4)	5 (3)

**Table 4:** Most frequent consumed food groups during the 24-h recall among the total sample (*n* = 354)

Food group	%
All starchy staple foods	99
White bread	87
Rice	57
Barley	36
Pasta	28
Flesh foods	78
Meat (cattle, camel, goat, sheep)	48
Fish (canned)	30
Chicken	20
Dairy	68
Milk (cow, camel, dry, goat, sheep)	67
Yoghurt	15
Cheese	13
Other vegetables	38
Onion	32
Tomato	30
Potato	31
Beans and peas	35
Other fruits	31
Apple	10
Pear	5
Other vitamin A-rich vegetables and fruits	21
Carrot	19
Pepper	5
Eggs	11
Nuts and seeds	3
Vitamin A-rich dark green leafy vegetables	**-**

### Meal pattern

The typical meal pattern consisted of three main meals: breakfast, lunch and dinner. Breakfast was consumed between 07.00 and 09.00 am and consisted of bread with oil, cheese or jam or the traditional dishes “azrig” (flour from maize or soya, liquid like yoghurt, milk or oil and sugar) or “ancha” (flour from barley, liquid and sugar). Most of the refugees also had traditional tea for breakfast. Lunch was normally eaten between noon and 3.00 pm and was mainly based on a hot dish consisting of vegetables with lentils, beans or bread as side dishes. Finally, dinner was mainly consumed between 9.00 and 11.00 pm and consisted of a larger meal of stew with vegetables and meat with rice, pasta or couscous as side dishes. Snacks, such as cookies and fruit and traditional tea were usually consumed throughout the day.

### Food rations

Of all the foods reported in the 24-h recalls, 67% was bought, 26% came from ration and 7% was received in the form of gifts or other. Seventy-seven percent reported that they did not receive enough dry foods and 74% reported that they did not receive enough fresh food in the food ration.

### DDS and socioeconomic determinants

Mean DDS was 3.1 (±1.2) for those with fewer than five assets, increasing significantly with number of assets to 4.4 (±1.4) for those with more than 9 assets (Table 5). Mean DDS for those with no education was 3.3 (±1.2) increasing with years of education to 4.7 (±1.5) for those with higher education (*p* < 0.001). Corresponding numbers when looking at women only were 3.6 (1.2) and 5.2 (1.3) (*p* < 0.001) respectively. In the group with no education, women had significantly higher DDS than men (p 0.036) while for the other educational levels no significant differences were found (data not shown). Mean DDS was not significantly different between participants with different types of latrine or income group. Further, mean DDS for different groups of WAMI score increased from 3.3 (±1.2) among those with the lowest WAMI (<7.5) to 4.3 (±1.4) in the group with the highest WAMI (>15) (*p* < 0.001) (Table 6). Finally, for groups of small WAMI index, mean DDS increased gradually from 3.1 (±1.2) among those with the lowest scores to 4.5 (±1.4) among those with the highest (*p* < 0.001).

**Table 5:** Distribution of sanitation, household assets, education and income and relation to dietary diversity (*n* = 355)

Number of assets *(0.5 p/asset* ^c^ *)*	Distribution, *n* (%)	Mean DDS [43]	
< 5	74 (21)	3.1 (1.2)	0.000^a^
5–6	93 (26)	3.8 (1.3)	
7–8	81 (23)	3.9 (1.2)	
≥ 9	107 (30)	4.4 (1.4)	
Education			
None *(0 p* ^*c*^ *)*	106 (30)	3.3 (1.2)	0.000^a^
1–6 grade *(2 p* ^*c*^ *)*	82 (23)	3.6 (1.3)	
7–9 grade *(4 p* ^*c*^ *)*	95 (27)	4.1 (1.4)	
10–12 grade (*6 p* ^*c*^ *)*	56 (16)	4.4 (1.4)	
Higher *(8 p* ^*c*^ *)*	16 (5)	4.7 (1.5)	
Sanitation			
No or Common latrine *0 p* ^*c*^ *)*	118 (33)	3.9 (1.3)	0.839^b^
Individual latrine *(4 p* ^*c*^ *)*	237 (67)	3.8 (1.4)	
Income			
No income *(0 p* ^*c*^ *)*	237 (67)	3.8 (1.4)	0.243^a^
1–30 Euro/mth *(4 p* ^*c*^ *)*	32 (9)	3.6 (1.2)	
31+ Euro/mth *(8 p* ^*c*^ *)*	78 (22)	4.0 (1.4)	
WAMI			
< 7.5	85 (24)	3.3 (1.2)	
7.5–10.5	85 (24)	3.9 (1.3)	
11.0–14.5	82 (23)	3.9 (1.3)	0.000^a^
≥ 15	89 (25)	4.3 (1.4)	

^a^Anova, significant if *p* < 0.05

^b^t-test for equality of means, significant if *p* < 0.05

^c^Indicates weights or points in the WAMI-index (maximum score is 28)

**Table 6:** Determinants of mean DDS among Saharawi refugee men and women (*n* = 355)

Dependent variables	Predictor variables	Unadjusted beta coefficients (95% CI)	*p*	Adjusted beta coefficients^a^ (95% CI)^b^	*p*	Stand Beta
Mean DDS	Constant					
	Age^b^	−0.02 (−0.03, −0.01)	*<0.001*	−0.01 (−0.06, −0.003)	***0.01***	−0.14
	Gender^c^	−0.40 (−6.0, −0.03)	0.11	−0.30 (−0.60, −0,04)	0.05	−0.11
	WAMI index^d^	0.07 (0.04, 0.10)	*<0.001*	0.07 (0.04, 0.100)	***<0.001***	0.31
	R^2^				0.14	

^a^MDDS adjusted for: sex, gender, number of children, marital status

^b^Continuous variable in years

^c^Dichotomous variable, 0 = women, 1 = men

^d^Continuous variable based on a score (WAMI index consists of 4 variables: water/sanitation, assets, maternal education and income)

A multiple regression model was used to identify determinants for mean DDS. In the final model, for every increase in mean DDS, the WAMI index increased with 0.07 (0.04, 0.10), *p* < 0.001. Age was negatively associated with DDS (*p* = 0.01). Men had −0.30 (−0.60, −0.04) lower mean DDS than women, *p* = 0.05 (Table 6).

## Discussion

In this study on dietary quality among adult Saharawi refugees, we found that only 1/3 of the refugees had adequate dietary diversity. Low socioeconomic status was associated with low DDS.

### Dietary diversity

Mean dietary diversity was 3.8 (±1.4) and only 32% of our sample had consumed foods from the minimum dietary diversity (MDD) threshold of five or more food groups [14] during the day of the interview. Thus, 2/3 of the Saharawi men and women probably are at risk of low dietary adequacy. Moderate to low DDS was also shown in the 2007 Food Security Survey, where the main score, based on a 7 day recall for 15–49 year old women, was 5.6 (±1.4) out of 11 food groups [16]. Despite different measurement periods, this implies that dietary diversity among women may have deteriorated. The 2012 Nutrition survey showed that overall 60% of households in the 5 camps had an adequate Food Consumption Score (FCS) (weighted household food group consumption in the past week), while 33% were borderline inadequate and only 7% had poor FCS [11]. On the first hand, this seems contradictory to our findings, and may reflect longer measurement periods, different cut-offs for adequacy or a more heterogeneous study population also including children and youth in the former study. On the other hand, it may also reflect poorer dietary quality in the camps in our study compared to 2012. Poor dietary diversity and low dietary adequacy has been shown in several studies from LMICs [17–21], but further direct comparisons are difficult because of the food aid received and the protracted environment in the refugee camps.

The multiple regression model showed no association between DDS and gender (p 0.05). While a study from Malaysia [22] showed that women had a higher Food Variety Score (FVS) (based on frequency of intake of food items during the previous week) than men, a study from Mali [2] showed that women were at higher risk of inadequate diets than men. Gender bias in intra-household food allocation has been thoroughly investigated, but based on a review of published literature, apart from in South-Asia, no clear gender preferences have been found [23]. Meanwhile, participation in household decision-making, has in a recent study been shown to be associated with higher DDS [24]. Saharawi households tend to be female-headed [25], which might influence food allocation and DDS. Further, the multiple regression model showed that DDS was negatively associated with age, comparable to the study from Mali [2]. Dietary diversity has previously been shown to be positively associated with higher energy intake [2, 26]. Lower DDS with increasing age may therefore reflect poorer appetite among older subjects [27, 28]. The coping mechanism most often mentioned among the refugees in the 2007 Food Security Survey was to reduce adult consumption so that children could eat [16]. It seems probable that food is allocated to the younger members of households at times of food scarcity, which might have a negative impact on DDS among older subjects. Finally, the positive association between dietary diversity and energy intake [2, 26] is reflected in previous studies reporting positive associations between DDS and body weight/BMI among Brazilian and Iranian women [29, 30]. Meanwhile, regression analysis showed no significant association between dietary diversity and BMI in our population, consistent with a recent meta-analysis of observational studies where no associations between DDS and BMI were found [31].

### DDS and socioeconomic determinants

Our study showed a positive association between DDS and SES, comparable to other studies from LMICs [2, 17, 32]. We assumed before our analysis that the refugees would be a homogenous group concerning SES given the protracted environment in which they live. In support of this we found that educational level was overall low, and 67% reported no fixed income at the time of the study. Using individual income may, however, be misleading since more than half (54%) of participants reported others in the household as their source of income, and 3% received money from relatives working abroad (data not shown). This may also have distorted associations between income and DDS in our study. Meanwhile, only 26% of the foods reported the previous day came from the food ration, which highlights the importance of family purchasing power. For instance, in the 2012 Joint needs assessment report it was found that for families in the lowest percentiles of FCS, the basic food ration accounted for up to 100% of consumption, while for families in the highest percentiles of FCS, the corresponding number was merely 40% [25]. Meat, milk and vegetables were among the products most commonly purchased [25]. Since these same products contribute significantly to improved DDS, this may in part explain the associations between SES and DDS.

Measuring socioeconomic status in low-income settings is often challenging due to monthly fluctuations in income and reporting bias. Attention has been drawn to supplement such data with household assets [15], a notion which seems relevant for our population where 25% in a previous survey [16] had savings listed as their primary source of income. Number of assets is a more direct reflection of accumulated household wealth than income [33] and has been linked to improved DDS in previous studies [3, 34]. Which socioeconomic factors contribute most to improved household diet is debated, where the weights have shifted from purely economic constraints towards more emphasis on maternal education and intelligence [35]. Associations between improved education [24, 36] or literacy [20] in women and DDS have been demonstrated in previous studies. Our data show a positive influence of education on dietary diversity even when the level of education in a population is moderate to low.

### Food rations

The high consumption of staple foods was expected based on what is received in the food ration and previous reports from the camps [16, 37]. Our study further supports earlier findings on high consumption of sugar and sugar-sweetened foods and drinks among Saharawi women, with a slight increase in consumption since 2007 [16]. Sugar was the commodity most commonly mentioned (by 74%) as insufficient in the food ration, followed by oil (68%)(data not shown), and this is supported by previous findings [25]. Finally, intake of flesh foods was common and the use of dairy seems to have increased since 2007 [16]. Even though the share of families with livestock (67%) increased since previous reports [16], a smaller proportion of the dairy consumed in our study (data not shown) was produced locally compared to 2007 [16]. Although meat and milk from sheep, goat and camel is part of traditional Saharawi food culture, and to some extent explain high intakes of flesh foods and dairy, the above changes may also be a result of the Nutrition transition with an increased consumption of western foods. Increasing prevalence of overweight and obesity among the Saharawi refugees, probably related to changing food habits and inactivity, has also been observed [13, 38]. While in studies conducted in 2007 [16] and 2008 [37], 55 and 75% of the refugees, respectively, had consumed vitamin A-rich fruits and vegetables the prevalence found in our study was only 20%. Thus, intake of certain fruits and vegetables appears to have decreased considerably.

Low dietary quality among the adult Saharawi refugees has many potential explanations and potential measures to improve dietary adequacy are numerous. The most obvious path is to investigate what is received in the food ration. Concern has been raised in recent years about funding and food security [39]. Our data underline the importance of keeping the basic food ration stable, while preferably increasing the distribution of fresh produce such as fruits and vegetables to increase dietary diversity, especially in families with low socioeconomic status. Owning livestock and home gardens has been associated with increased food security and dietary diversity in other settings [20, 40], but DDS did not differ significantly between participants with or without livestock and/or vegetable gardens in our study. These gardens have for the most part been unsuccessful in the camps because of the salty soil and harsh climate [25]. Since a high proportion of foods (67%) was purchased, our data suggest that the Saharawi refugees are highly dependent on supplying the basic food ration with commodities from local stores. Targeted voucher or cash programs to cover basic food and non-food items have been suggested [25]. Based on our data it seems to be a viable option to improve DDS in the Saharawi refugee camps.

### Strengths and limitations

The strength in this study was a large sample size, covering a considerable age span and both genders. Concerning methodology, dietary diversity score is simple to use [41] and adaptable to different settings [42]. Further, it is recommended when a common food bowl is used [3], such as Saharawi food culture, where estimation of quantities may be challenging. However, different application of number of food groups, length of reference periods, and use of minial intake limits in previous studies makes comparison of findings difficult. Finally, since the food ration does not cover 30 days for most commodities [25], the timing of the interview might strongly influence DDS for those fully dependent on the basic food ration, and data on sources of food (ration or purchased) in general.

## Conclusion

We found that only 1/3 of the refugees had adequate dietary diversity. Thus, the Saharawi men and women probably are at risk of low dietary adequacy. Low socioeconomic status seemed to be associated with low dietary diversity score. Our findings may be used by local health authorities or international donors as knowledge base for future targeted interventions to improve dietary adequacy among the Saharawi refugee population.

## Additional file

Additional file 1: Table S1. Ownership of assets and dietary diversity. (PDF 1003 kb)

## Abbreviations

BMI: Body mass index
DDS: Dietary diversity score
FANTA: Food and Nutrition Technical Assistance Project
FAO: Food and Agriculture Organization of the United Nations
FCS: Food consumption score
FVS: Food variety score
LMIC: Low- and middle income country
MDD: Minimum dietary diversity
MDDS: Mean dietary diversity score
MDD-W: Minimum Dietary Diversity – Women
OpenEpi: Open Source Epidemiologic Statistics for Public Health
PPS: Probability proportional to size
SES: Socioeconomic status
WAMI: Water/sanitation, Assets, Maternal education, Income
