# Supplementary material for: Dietary diversity is related to socioeconomic status among adult Saharawi refugees living in Algeria
Source: BMC Public Health. 2017 Jul 3;17:621. doi: 10.1186/s12889-017-4527-x (PMC5496305; doi:10.1186/s12889-017-4527-x)
Supplement: Additional file 1: Table S1. — Ownership of assets and dietary diversity. (PDF 1003 kb) [file 12889_2017_4527_MOESM1_ESM.pdf]

Table 1 (supplement) Ownership of assets and dietary diversity

Characteristic	% that have asset (n=355)	Mean DDS [43] Without asset	Mean DDS [43] With asset	*P*[1]
**Assets items**				
Kitchen	96	3.6 (1.5)	3.8 (1.4)	0.475
**Cell phone**	**80**	**3.3 (1.3)**	**4.0 (1.3)**	**0.000***
Solar energy	78	3.8 (1.4)	3.9 (1.4)	0.591
**TV**	**68**	**3.5 (1.3)**	**4.0 (1.4)**	**0.002***
**Oven**	**66**	**3.4 (1.3)**	**4.1 (1.3)**	**0.000***
**Refrigerator**	**51**	**3.6 (1.4)**	**4.1 (1.3)**	**0.000***
**Converter**	**50**	**3.7 (1.3)**	**4.0 (1.4)**	**0.019***
**Air condition**	**46**	**3.7 (1.3)**	**4.1 (1.4)**	**0.004***
**Radio**	**42**	**3.6 (1.4)**	**4.1 (1.3)**	**0.002***
**Sitting furniture**	**38**	**3.7 (1.4)**	**4.1 (1.3)**	**0.001***
**Car**	**34**	**3.7 (1.3)**	**4.0 (1.4)**	**0.045***
**Washing machine**	**21**	**3.7 (1.3)**	**4.3 (1.4)**	**0.002***
**Sleeping mattress**	**11**	**3.8 (1.3)**	**4.4 (1.5)**	**0.006***
**Laptop**	**7**	**3.8 (1.3)**	**4.7 (1.6)**	**0.001**
Fan	6	3.8 (1.4)	4.2 (1.5)	0.177
Aggregate	3	3.8 (1.4)	4.1 (1.4)	0.549

[1]t-test for equality of means, significant if p<0.05
